# Parasitism by Entomopathogenic Fungi and Insect Host Defense Strategies

**DOI:** 10.3390/microorganisms13020283

**Published:** 2025-01-27

**Authors:** Dinghai Zhang, Haidi Qi, Feng Zhang

**Affiliations:** 1 Centre for Quantitative Biology, College of Science, Gansu Agricultural University, Lanzhou 730070, China; qhd17539@163.com; 2 CAS Key Laboratory of Tropical Forest Ecology, Xishangbanna Tropical Botanic Garden, Chinese Academy of Sciences, Mengla 666303, China; fzhang@xtbg.ac.cn

**Keywords:** infection mechanisms, insect defense strategies, immune response, co-evolution, disease dynamics

## Abstract

Entomopathogenic fungi, a group of insect pathogens, are characterized by high insecticidal efficacy and minimal environmental impact. They are commonly used as biopesticides for pest control due to their significant practical value. We here classify entomopathogenic fungi according to the process of fungal infection in hosts, changes in host behavior during infection, and mechanisms of spore transmission, and review the strategies employed by insects to resist infection, including physical barrier defenses, immune system defenses, and behavioral avoidance of fungal pathogens. This review also discusses the pathogenic mechanisms of fungi on insects and the closely linked co-evolution between fungal pathogens and insect defenses. In conclusion, a perspective on future research is provided, emphasizing the impact of insect population density and spore concentration in the environment on disease outbreaks.

## 1. Introduction

The global population is projected to rise from 7.4 billion in 2016 to 8.1 billion by 2025, with estimates suggesting it could reach nearly 9 billion by the mid-21st century [1]. This growth, alongside increasing food consumption, has put immense pressure on agricultural systems worldwide. In this context, arthropod pests are estimated to cause annual crop yield losses of 18% to 26%, significantly impacting food security [2]. As agricultural productivity needs to meet the demands of a growing population, the issue of pest control has become increasingly urgent. Traditional reliance on chemical pesticides has introduced several challenges, including adverse effects on non-target organisms, groundwater contamination, pesticide residues, and the development of pest resistance [3,4]. These concerns highlight the need for more sustainable pest management strategies.

Entomopathogenic fungi present a promising solution in this regard. These fungi offer a more eco-friendly alternative to chemical pesticides, promoting sustainable agricultural practices while also reducing the economic burden associated with conventional pest control methods. Unlike chemical pesticides, entomopathogenic fungi have minimal environmental impact, are cost-effective, and exhibit a lower tendency to induce resistance in target pests. Furthermore, they are harmless to humans and non-target organisms, making them an ideal tool for integrated pest management [5,6]. Recent advances have further enhanced the potential of entomopathogenic fungi, with the discovery of more virulent strains and the use of new application techniques such as nanomaterials. Moreover, research on fungal infection mechanisms at the molecular level, as well as the development of genetically engineered fungi, has further boosted their efficacy as biopesticides [6,7].

The use of entomopathogenic fungi in pest control is not a novel concept. Early research, dating back to the 19th century, focused on their potential to control diseases in the French silk industry. In 1835, Agostino Bassi identified the fungus *Beauveria bassiana* (Pierre-Marie-Alexis Beauverie) as the cause of muscardine disease in silkworms, marking the first recognition of an insect-pathogenic fungus [8]. This discovery laid the foundation for the use of entomopathogenic fungi as biocontrol agents. Later, in 1888, Elie Metchnikoff furthered the field by testing fungi isolated from beer mash on field crops, demonstrating their potential for pest management [9]. These early studies catalyzed the development of entomopathogenic fungi as a viable biopesticide, an innovation that continues to shape modern agricultural practices.

Recent research has explored the genomic and biochemical factors that determine the pathogenicity of entomopathogenic fungi. For instance, genome sequencing of *B. bassiana* and *Metarhizium anisopliae* (Andreas Franz Wilhelm Möller) has revealed specific genes associated with pathogenicity, aiding in the development of more efficient fungal strains for pest control [10]. Furthermore, secondary metabolites produced by fungi have been found to interfere with insect immune responses, opening up new avenues for enhancing fungal virulence. The application of genetically engineered fungi, such as those producing insecticidal proteins or exhibiting higher host specificity, has also garnered significant attention. Additionally, researchers have focused on optimizing application methods, such as the use of microencapsulated or spore-embedded formulations, which can improve the persistence and effectiveness of fungi under field conditions.

This article aims to provide an in-depth review of the interaction between entomopathogenic fungi and their host insects, focusing on the mechanisms of fungal infection, insect defense strategies, and the influence of fungal pathogens on host behavior. Additionally, the article discusses the interaction between fungal infections and host immune responses and offers insights into future prospects, including mathematical models for disease transmission.

## 2. The Classification of Entomopathogenic Fungi

Entomopathogenic fungi induce mortality in their hosts by affecting developmental and reproductive processes. Currently, 750–1000 fungal species have been identified as pathogenic to insects [7], commonly belonging to the phyla *Ascomycota*, *Basidiomycota*, *Chytridiomycota*, *Glomeromycota*, *Mucoromycota*, and *Entomophthoromycota* [11,12]. The phylum *Entomophthoromycota* was formerly classified within the phylum *Basidiomycota* until its elevation to an independent phylum [13]. Fungal hosts are prevalent across nearly all insect orders, with the most common being *Coleoptera*, *Diptera*, *Hemiptera*, *Lepidoptera*, *Orthoptera*, and *Hymenoptera*. The entomopathogenic fungi mentioned in this article and their classifications are listed in Table 1. It is noteworthy that some fungi have limited host ranges; for example, *Aschersonia aleyrodis* (Carlos Emilio S. Hennings) infects only whiteflies [14], while *Metarhizium rileyi* (J. M. McCoy) infects only *Lepidoptera* and *Coleoptera* [15,16]. However, *B. bassiana* and *M. anisopliae* have a broad host range and strong pathogenicity, capable of infecting over 700 insect species across multiple orders, making them versatile and effective for pest management. Their ability to survive in diverse environmental conditions further enhances their application potential. Additionally, they are safe for non-target organisms, making them ideal candidates for sustainable pest control in agriculture [17].

The host specificity of entomopathogenic fungi presents both challenges and opportunities in the application of biological pesticides. Fungi with narrow host ranges can be highly effective for controlling specific pest species. However, their limited host range also restricts their broader application in pest management. For manufacturers of biological pesticides, fungi with strong host specificity may not meet the diverse pest control needs of different agricultural systems, leading to increased costs and complexity in their use. Nevertheless, the host specificity of certain entomopathogenic fungi can also be seen as an opportunity. This specificity reduces the risk of harming beneficial organisms such as pollinators and natural predators, allowing for more sustainable pest management practices, particularly in systems where only a particular pest is of concern. Thus, while host specificity may limit the versatility of some fungal species, it also enhances their ecological safety and precision in pest control, making them a valuable tool for sustainable agriculture.

## 3. The Mechanisms by Which Entomopathogenic Fungi Infect Their Hosts

### 3.1. Direct Mortality

#### 3.1.1. Epidermal Adherence

Entomopathogenic fungi exist in the form of spores on plant surfaces, soil, water, or in the air. Unlike bacteria or viruses, entomopathogenic fungi do not require ingestion by insects; instead, they can directly penetrate the host’s intact cuticle to access the hemocoel [18,19]. Fungal adhesion to the cuticle is a multifaceted process that primarily involves passive attachment mediated by non-specific hydrophobic and electrostatic forces, alongside specific ligand-receptor interactions [20,21]. Both *B. bassiana* and *M. anisopliae* are capable of producing hydrophobic conidia, which are equipped with a surface rodlet layer composed of hydrophobic proteins, such as hydrophobin, that play a critical role in facilitating the initial attachment to both hydrophobic and hydrophilic surfaces. These proteins are characterized by their high content of nonpolar amino acids, which enable them to form a protective hydrophobic coating around the conidia. This coating promotes tight adhesion to the insect cuticle by engaging in hydrophobic interactions with the surface and by forming transient electrostatic bonds with the cuticular chitin. Structurally, hydrophobic proteins typically contain multiple *β*-sheet and *α*-helix regions that contribute to their stability and function in different environmental conditions. This structural configuration not only helps the spores resist environmental stresses but also facilitates their interaction with host surfaces, thus enhancing the likelihood of successful infection.

Additionally, specific adhesins, including the characterized proteins MAD1 and MAD2, are involved in the fungal adherence process. These adhesins interact with host cell receptors, directly mediating the attachment of conidia to the host and contributing to the subsequent fungal colonization. The production of these adhesins is regulated at the transcriptional level, and their expression is critical for the fungus’s ability to penetrate the cuticle and establish infection [22].

#### 3.1.2. Cuticle Penetration

**Figure 1:**
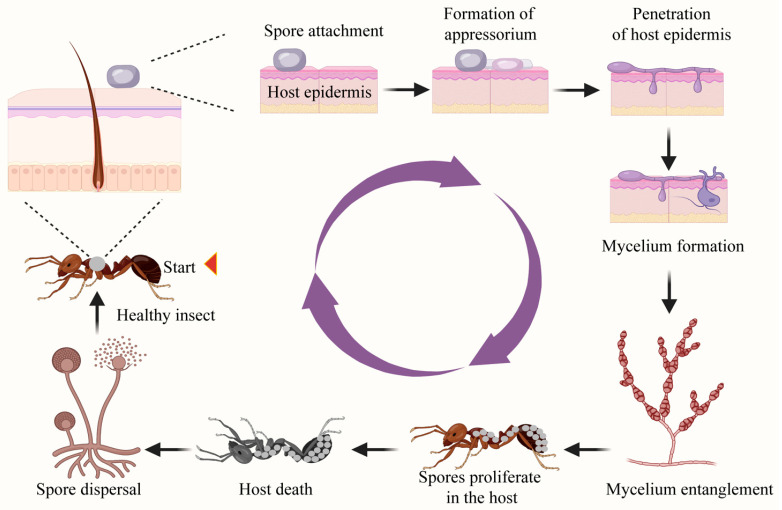
The mechanisms of entomopathogenic fungi invading hosts. The figure depicts the infection process of entomopathogenic fungi in insects. Fungal spores attach to the insect’s surface, form appressoria to penetrate the cuticle, and develop mycelium inside the host. As the fungus spreads, it kills the host, proliferates, and produces new spores, which are then dispersed to infect other insects, completing the infection cycle.

Germinating spores produce germ tubes with appressoria or penetration pegs (Figure 1), exerting strong mechanical pressure on the insect’s cuticle [23]. Appressoria are specialized structures formed by fungi on the host surface, enhancing adhesion and applying penetrating pressure to help the fungus breach the host’s protective outer layer. Appressoria contain abundant Golgi bodies, mitochondria, ribosomes, and endoplasmic reticulum. Golgi bodies are responsible for processing and packaging proteins, assisting in their secretion or transport to other cellular locations and the endoplasmic reticulum participates in protein synthesis and transport. These organelles synthesize and secrete a large number of degradative enzymes, such as proteases, lipases, and chitinases, which help break down the host’s cuticle, allowing the fungus to penetrate under enzymatic and physical pressure [24,25]. Additionally, spores can also enter the insect’s body through openings such as spiracles, mouthparts, and wounds [26].

#### 3.1.3. Proliferation and Toxin Metabolism

Upon successful adhesion and penetration, fungi consume the host’s nutrients to sustain their own proliferation and disrupt the host’s original tissue structure. Following cuticle penetration, the fungi typically proliferate in the hemocoel in the form of yeast-like budding spores or hyphal bodies (Figure 1). This growth is regulated by the host environment and is accompanied by the secretion of enzymes, such as chitinases and proteases, which break down host tissues and facilitate further fungal invasion. Concurrently, the fungi produce secondary metabolites, including a variety of toxins such as destruxins, beauvericin, bassianolide, and peptide toxins, which severely compromise the host’s immune system [27,28]. These metabolites not only hinder the immune response but also contribute to tissue damage and host immunosuppression. Common toxins such as destruxins inhibit host cellular signaling pathways, leading to apoptosis and immune evasion, while beauvericin disrupts mitochondrial function, impairing energy production in the host [29]. Infected insects may succumb to the effects of nutrient depletion, extensive tissue damage, or disruption of normal physiological functions caused by spore growth and the toxic substances released by the fungus.

#### 3.1.4. Spore Dispersal

The spores of entomopathogenic fungi can erupt spontaneously from insect cadavers, propelled distances up to 1000–1500 times their own diameter, or, in the case of *Entomophthora muscae* (Herman Rudolf Hesse), distances of approximately 5–10 mm, dispersing into the air and onto plant surfaces surrounding the cadaver [18,30,31]. Many fungi in the order *Hypocreales* and *Entomophthorales* can produce resting spores, which are not infective themselves but can survive for long periods in the absence of a host [32,33]. Under favorable conditions, they germinate to produce infective spores or directly infect hosts (Figure 1).

### 3.2. Control of Host Behavior by Entomopathogenic Fungi

The diversity of fungal species correlates with significant variations in host range. Many fungi within the genera *Beauveria* and *Metarhizium* exhibit broad host ranges, rapidly consuming and killing their hosts within days of infection without inducing significant behavioral changes [34,35]. Conversely, numerous fungi within the orders *Hypocreales* and *Entomophthorales* possess the ability to manipulate host behavior [36]. Alterations in host behavior constitute a critical factor influencing fungal dissemination. Fungal manipulation of host behavior enhances the efficiency of spore dispersal, thereby increasing their own survival capabilities. Host manipulation typically involves a series of aberrant behaviors, including aimless wandering and climbing onto plants at the end of infection to facilitate spore ejection from higher positions, often accompanied by phototactic responses. Upon reaching their final location, controlled insects are often induced to exhibit gripping behavior to ensure adherence to the underlying substrate. Additionally, infected insects may spread their wings to better facilitate spore discharge. These manipulative behaviors are not uniformly expressed throughout the day, occurring only at specific times. Insects infected with Entomophthora fungi also exhibit heightened mating behavior, increasing spore dissemination through direct contact.

Behavior manipulation not only facilitates fungal transmission but also provides new insights for pest management strategies. By manipulating host behavior, pathogenic fungi can increase pest mortality without relying on chemical pesticides, thus reducing the negative impact on the environment and non-target species. This behavioral manipulation mechanism offers the potential for developing eco-friendly pest control methods. Further research could reveal the specificity of different fungi in manipulating host behavior, laying the foundation for the design of behavior-based biological control strategies.

#### 3.2.1. Fungal-Induced Hyperactivity

Entomopathogenic fungi rely on the movement of hosts to transport them to environments conducive to spore production and dissemination. For instance, *Massospora cicadina* (Charles H. Peck) produces infective spores while the host cicada is still alive and maintains the host’s activity to promote spore dispersal [37]. Ants infected by *Ophiocordyceps unilateralis* (Jean-Michel P. M. Candido and J. H. S. De Lemos) exhibit aimless wandering behavior and display unstable movements and tremors when leaving the nest to climb onto plants. They frequently collapse due to convulsions, allowing the infected individuals to leave the ant colony and climb onto nearby plant stems or branches before being noticed by other colony members, which is crucial for the survival and reproduction of the fungus [38,39]. Because healthy ants actively attack infected individuals as part of their social immune strategy, this interferes with the formation and dissemination of final spores [40]. Therefore, host activity can also be viewed as a strategy for fungal spore dissemination after the death of the host.

#### 3.2.2. Summit Behavior in Infected Insects

The aimless wandering activity of infected insects contributes to inducing what is known as “summit behavior” in insects, which involves climbing to relatively higher positions as death approaches. Many insects infected by fungi from the orders *Hypocreales* and *Entomophthorales* display climbing behavior towards elevated locations [41,42]. Ejecting spores from elevated positions allows for better utilization of wind for spore dispersal, while also enabling insects to distance themselves from attacks by conspecifics and be situated in environments with suitable temperature and humidity for spore germination [43]. In indoor environments, insects may die at the tops of windows and walls or cling to eaves [44]. In open areas, insect mortality typically occurs at the tops of plants, 5–80 cm above the ground. Climbing behavior of insects can also be observed in wire cages where temperature does not vary with height; for instance, house flies (*Musca domestica*) infected with *E. muscae* will climb to the top of the cage and die on the ceiling and suspension lines [45]. Although death at higher positions facilitates spore dispersal, it also increases the likelihood of insects being preyed upon by natural enemies and other predators, making it difficult to determine whether “summit behavior” benefits the insects or the fungal pathogens [46].

#### 3.2.3. Death Grip and Fixation in Infected Insects

Infected insects typically exhibit additional adaptive behaviors after climbing to the top of plants, such as producing hyphae and fixing their bodies to the substrate using the sticky substance secreted by the hyphae. The insects, thus immobilized, can remain attached to surfaces like leaves, branches, or even window glass for several days to weeks after death. This behavior, where the insect tightly grips the substrate even in death, is also referred to as “death grip” [46,47,48]. Fungi immobilize insects in a particular location to increase the likelihood of new hosts coming into contact with spores through feeding, resting, or mating behaviors. Behavior involving attachment via pseudoroots has been observed in aphids and ants infected by Pandora fungus [30]. Some fungi do not produce pseudoroots, and the host relies solely on its own structures (such as legs or mouthparts) for fixation. In many insects infected by entomopathogenic fungi, legs serve as important anchoring tools. Grasshoppers infected with *Entomophaga grylli* (Augustus B. Baker), upon reaching the top of herbaceous plants, exhibit bent legs, with the forelegs and midlegs tightly grasping the plant before succumbing to death [49]. In fireflies infected by entomopathogenic fungi, fixation by biting vegetation with mandibles has been observed. After being fixed, some insects raise their abdomens while spreading their wings in a posture resembling “launching”. Soldier beetles (*Chauliognathus pensylvanicus*) infected by *Eryniopsis lampyridarum* (Pierre-Michel Gauthier and Jacques S. P. G. R. Moreau) exhibit an unusual posture where their bodies tilt upward at a 45-degree angle, with mandibles tightly gripping the calyx and wings raised as if in flight. This unusual posture is also an expression of fungal control over host behavior, facilitating increased distance for spore release [46,50]. The “biting behavior” of ants infected by *O. unilateralis* is another notable example, where the fixation mechanism is similar to soldier beetles; the infected ant’s mandibular muscles contract excessively, gripping tightly onto branches or leaf veins [51].

#### 3.2.4. Circadian Rhythm of Host Mortality

Many studies indicate that the time of host mortality by many fungi in *Entomophthorales* and *Hypocreales* exhibits a circadian rhythm [52]. Ants infected with *O. unilateralis* exhibit leaf-biting behavior around midday, clinging to the leaves they bite for up to 6 h before perishing in the evening [38] Similarly, flies infected with *E. muscae* have been observed to die at dusk [53]. Soldier beetles infected with *E. lampyridarum* typically perish in the early morning, but it is not until the following evening that spore eruption from the thoracic and abdominal regions occurs [46]. These examples all suggest that fungal-induced mortality in hosts occurs at specific times of the day, possibly due to the more favorable conditions for spore formation during humid nights.

#### 3.2.5. Rampant Mating Behavior Induced by Infection

Fungi can alter the appearance of infected insects to make them more attractive or secrete chemicals to enhance insect excitability. This increases the sexual attractiveness of infected insects to conspecifics, inducing mating behavior through direct contact to promote spore dissemination. The size of the abdomen is a crucial factor in attracting male flies for mating, as larger abdomens in female flies typically lead to greater egg production. Due to the growth of fungal hyphae, the abdomens of houseflies infected with *E. muscae* significantly swell, attracting healthy houseflies to the abdominal features of infected cadavers. Male housefly cadavers are often more appealing, possibly due to their larger abdomens [30,54]. Experimental evidence indicates that when the abdomens of infected and uninfected houseflies are of equal size, infected housefly cadavers still exhibit greater attractiveness than uninfected ones, potentially due to the fungus secreting a chemical attractant for males [30]. Studies show that cicadas infected with *M. cicadina* contain psilocybin and cathinone in their bodies, both hallucinogens and stimulants that induce hyperactivity and increased mating desire in infected cicadas [55,56]. The terminal abdominal segments (including the reproductive organs) detach in infected cicadas, with large masses of fungal spores appearing at the rupture site [37,57]. Infected cicadas can still fly, expelling spores from the abdomen and emitting mating calls. Infected females even respond by wing flicking to male calls. Even cicadas with half of their abdomen missing engage actively in mating behavior, often observed attaching to remnants of the abdomen torn off during mating from their partners [56].

## 4. Insect Defense Strategies

The defense mechanisms of insects share many common features with the innate immune system of vertebrates, and significant achievements have been made in the study of insect immunity [58]. To combat infection, insects have evolved various defense strategies, including physical barriers through the cuticle and their own immune system, behavioral immunity by seeking high-temperature environments to raise body temperature and avoid infected individuals, as well as social immunity in gregarious insects, which involves timely removal of infected individuals’ corpses from the nest, nest sanitation, and mutual grooming for body hygiene.

### 4.1. Defense Mechanisms

The insect cuticle serves as the first physical barrier against fungal infection. Structurally, the insect cuticle consists of the epicuticle, procuticle, and epidermis, arranged from the inside out. The epidermis, in turn, is further divided into inner, outer, and surface layers, with the surface layer comprising the epicuticle, wax layer, and cuticular layer. For pathogenic fungi, penetrating through the complex multilayered structure of the insect cuticle is exceptionally challenging [59,60]. Moreover, the physiological environment of the cuticle exerts inhibitory effects on spore germination: it has low water activity, limited nutrients, and contains antimicrobial compounds. Among these, the hydrophobic lipids in the epicuticular wax layer and antimicrobial compounds secreted by the host or resident microbes can inhibit spore attachment and germination [61,62,63]. For instance, *Bacillus pumilus*, residing on the cuticle of the corn leafhopper (*Dalbulus maidis*), inhibits the germination of *Beauveria* spores. Additionally, some social insects secrete antimicrobial substances to prevent fungal infections and disease transmission. Ants’ metapleural glands secrete antibiotics to counteract fungal infections [64].

The epidermis initiates its defense by secreting fungal protease inhibitors and synthesizing melanin in epidermal cells. One example is the insect metalloproteinase inhibitor, which is involved in conferring resistance against insect-pathogenic fungi-specific metalloproteinases [65]. During fungal penetration of the corneous layer, phenoloxidase in epidermal cells catalyzes the synthesis of melanin [65,66]. Melanin and its precursors not only restrict fungal growth but also inhibit the synthesis of epidermal-degrading enzymes, playing a crucial role in protecting the insect cuticle from fungal invasion [67].

Once fungi successfully enter the insect hemolymph, they proliferate in the form of yeast-like budding spores or hyphal bodies and colonize within the hemocoel. Research has revealed an insect defense strategy involving the induction of cross-kingdom silencing of pathogenicity-related genes using miRNA, thereby conferring insect resistance to infection. Upon penetration of *B. bassiana* into the host hemocoel, the expression of let-7 and miR-100 microRNAs (miRNA) increases in mosquitoes. These miRNAs translocate into the fungal hyphae and specifically silence the sec2p and C6TF genes associated with fungal virulence, which encode Rab guanine nucleotide exchange factor and Zn(ll)2Cys6 transcription factor, respectively [68].

### 4.2. Insect Behavioral Immunity to Fungal Infection

In addition to physical and immune defenses, insects employ behavioral strategies to avoid or mitigate the effects of fungal infections. Insect behavioral immunity to fungal infections appears to be influenced by the nature of the fungal pathogen, with certain strategies proving more effective against specific fungal species. This variation in defense effectiveness can be attributed to differences in fungal virulence, infection strategies, and environmental conditions that favor particular types of fungi. Behavioral fever, as observed in locusts and houseflies, seems particularly effective against entomopathogenic fungi that rely on environmental conditions for spore development, such as *Entomophaga grylli* and *Metarhizium* species. These fungi are highly sensitive to temperature fluctuations, and the behavioral fever strategy significantly inhibits spore development and reduces infection rates [69,70]. For instance, locusts infected with *E. grylli* have been observed to climb to higher, sunlit areas where temperatures exceed 35 °C, significantly reducing fungal spore development and mortality rates [71]. Experimental data demonstrate that locusts exposed to temperatures between 35 °C and 40 °C have their fungal development inhibited, with a reduction in mortality by approximately 40%. Similarly, houseflies, when infected, survive longer when exposed to temperatures above 40 °C, with survival time extended by about 50%, while fungal development is significantly suppressed [72,73]. In contrast, fungi that produce more resilient spores or those capable of adapting to a wide range of environmental conditions may not be as easily suppressed by behavioral fever.

Another key behavioral strategy is escaping behavior, where infected insects release alarm signals to warn others of the infection. Termites infected by *M. anisopliae* exhibit body vibrations to alert healthy individuals, prompting them to avoid the infected ones. This response is critical in limiting the spread of infection within the colony [74]. However, as seen in aphids infected by *Pandora neoaphidis* (L. M. Humber), the ability to respond to alarm pheromones may be diminished, making them more susceptible to predation and reducing the density of fungal spores in the environment [75].

### 4.3. Insect Social Immunity Against Fungal Infection

Social insects, such as ants and bees, have evolved complex collective strategies to resist fungal infections. One key behavior is the removal of diseased individuals. For example, ants infected with *O. unilateralis* fungus quickly remove infected corpses before fungal spores are produced. Research shows that ant colonies clear infected bodies within 24 h of detection, preventing fungal spore formation and transmission [76]. Furthermore, removing infected corpses reduces the fungal spore density within the colony, lowering the overall infection rate by 30–50% [77,78]. Similarly, bees exhibit similar behavior to ants by promptly removing infected larvae, thereby preventing the spread of *Ascosphaera apis* (C. T. Ingold) spores. Studies have shown that bees are able to remove up to 80% of infected larvae within 48 h, significantly reducing the spread of the fungal infection within the hive [79,80]. Bees with stronger hygienic behavior tend to detect infection earlier and remove larvae before the spores become highly infectious [81]. There is a negative correlation between the proportion of hygienic bees in a colony and the infection rate, with colonies exhibiting more hygienic behavior showing significantly lower infection rates. However, this strategy may not be as effective against fungi that have evolved mechanisms to avoid early detection or that do not produce visible signs of infection, such as *Batrachochytrium dendrobatidis* (Matthew B. Berger)*,* which infects amphibians [82]. In these cases, the infection may go undetected for longer periods, and the removal of infected individuals may be less frequent, reducing the overall effectiveness of the behavior.

In addition to corpse removal, social insects also engage in nest sanitation and cleaning behavior. For instance, ants use antimicrobial resin collected from their environment to disinfect their nests [83]. Furthermore, ants secrete formic acid and other acidic substances that can kill fungal spores, which enhances the antifungal activity of the resin, protecting the ants from infection [84]. Self-grooming and mutual grooming behaviors also play an important role in reducing fungal burden within the population. However, despite its benefits, grooming may also facilitate the transmission of spores between individuals.

## 5. The Interaction Between Fungal Infection and Host Insect Immunity

Infection of host insects by pathogenic fungi leads to significant changes in host behavior, primarily mediated through chemical signaling between the fungus and the host’s neuro-muscular system. Fungi release chemical signals that deplete the host’s nutrient reserves or modulate the host’s physiological functions, thereby altering host behavior. For example, depletion of nutrient reserves by the fungus induces a state of hunger in the host, prompting behavioral changes to replenish internal nutrient stores. Genomic and transcriptomic studies have revealed changes in the expression levels of hunger-related genes in ants infected by *O. unilateralis* [85]. Additionally, upregulation of genes encoding small secreted proteins (SSPs) has been observed when hosts exhibit biting behavior, suggesting that these SSPs may contribute to changes in host behavior [86,87]. Furthermore, compounds such as guanidinobutyric acid and sphingosine, which affect neural function, have been found in the brains of *Camponotus castaneus* ants infected by *O. unilateralis*, indicating that fungal metabolites can influence the host’s brain even without direct contact between the fungus and the host’s brain [88,89]. These findings provide insight into how entomopathogenic fungi manipulate host behavior through chemical signaling and the molecular mechanisms that may underlie this process.

However, insects are not entirely defenseless against these pathogenic fungi. In response to fungal infections, insects rely not only on behavioral avoidance but also on physical barriers, such as the integument, and innate immune defenses. The pathogenic mechanisms of entomopathogenic fungi are closely linked to the host’s immune system, and the two interact in a dynamic co-evolutionary process. In interacting populations, individuals can alter their genetic structures in response to changes in the traits of the other. Entomopathogenic fungi have evolved numerous strategies to overcome insect defense systems, while insects continue to evolve new mechanisms to resist fungal infections. For example, insects rely on broad-spectrum antimicrobial peptides (AMPs) to resist fungi. To counteract the insect-induced synthesis of AMPs, fungal pathogens have evolved the ability to suppress the host’s innate immune response [90,91]. In addition to inhibiting the synthesis of AMPs, entomopathogenic fungi can produce virulence-associated proteases that degrade host defense molecules, including AMPs, and inhibit the host’s cellular immune response [91,92]. These proteases can co-evolve with insect protease inhibitors, which are part of the host’s immune arsenal. Fungal proteases are recognized as virulence factors capable of penetrating the insect cuticle and undermining the host’s immune defenses.

*Galleria mellonella* produces three serine protease inhibitors that are induced and secreted into the hemolymph, along with AMPs, to inhibit fungal infection. Studies have shown that only proteases that cannot be fully neutralized by host protease inhibitors in the integument, tissues, and hemolymph can act as fungal virulence factors [91]. This indicates that fungi evolve to produce proteases that are not susceptible to host inhibitors, ensuring that these proteases remain effective at low concentrations. For instance, metallo-proteases from the thermolysin family exhibit such properties. Insects typically do not produce specific inhibitors of metalloproteases, allowing these enzymes to act as virulence factors [93].

A prominent example of co-evolution between fungi and insect hosts is seen in the interaction between *M. anisopliae* and *Spodoptera* species. *M. anisopliae* is a widespread entomopathogenic fungus, and its interaction with insect immunity is highly dynamic. Research has shown that *Spodoptera* species exhibit significant immune responses to *M. anisopliae* infection, including the synthesis of AMPs, activation of peroxidase enzymes, and reinforcement of mechanical barriers. In response, *M. anisopliae* has evolved various immune-suppressing factors, known as fungal immune suppressors (FIS), which inhibit the host’s immune response, allowing the fungus to penetrate the insect’s cuticle and spread within the host [94]. However, insects do not passively succumb to fungal infection. Over time, insect hosts have evolved enhanced resistance to fungal pathogens. For instance, *Spodoptera* species increase the production of AMPs and enhance physical barriers, such as thickening the cuticle, in response to repeated fungal infections [95]. This process highlights the arms race between the insect’s immune defenses and the fungal pathogen, where both the host and the pathogen evolve in response to each other’s adaptations. This co-evolutionary dynamic drives the development of increasingly sophisticated immune strategies in insects and immune suppression mechanisms in fungi, thereby shaping the interactions between the two.

These studies clearly illustrate the co-evolutionary process between entomopathogenic fungi and insect hosts, emphasizing how fungal adaptations drive changes in insect immunity and vice versa. Fungi evolve immune-suppressing factors, virulence factors, and other mechanisms to overcome the host’s immune defenses, while insects develop new immune strategies to counteract fungal infection.

## 6. Prospects

### 6.1. Actionable Recommendations to Overcome Limitations of Entomopathogenic Fungi

Despite the considerable potential of entomopathogenic fungi as biological control agents, they face several limitations, including environmental sensitivity, high storage costs, and host specificity [96,97]. To effectively address these challenges, we propose actionable recommendations: (1) Developing fungal strains or formulations with enhanced resilience to environmental stressors, such as UV protection and temperature stabilization, can improve their field efficacy. (2) Researching cost-effective production and storage methods will help lower the expenses associated with commercial formulations, increasing their accessibility for agricultural use. (3) Explore genetic engineering approaches to enhance the virulence and environmental tolerance of these fungi, potentially broadening their effectiveness against a wider range of pests. (4) Investigating the synergistic use of entomopathogenic fungi with other biocontrol agents, such as parasitoids or beneficial microorganisms, could lead to integrated pest management strategies that enhance overall efficacy. (5) Conducting thorough surveys to assess pest prevalence and resistance patterns prior to deploying entomopathogenic fungi will ensure the selection of appropriate species for targeted pest control.

### 6.2. Mathematical Modeling of Fungal Infections in Host Populations

Currently, research focuses mainly on the molecular mechanisms of entomopathogenic fungi infecting host insects, with a lack of studies from the perspective of mathematical models on when fungal-induced infections can invade host populations. Understanding how pathogens invade hosts and cause transmission is the foundation of infectious disease research [98]. Future research can be conducted based on the transmission mechanisms of entomopathogenic fungi in host populations and dynamic models of infectious diseases.

Establishing differential equation models concerning the susceptible, infected individuals, and the change of spore concentration in the environment over time. Here, susceptible individuals represent hosts that are currently uninfected but lack immunity and can become infected, while infected individuals have already been infected and can transmit the disease to susceptible individuals. Each infected insect releases a large number of spores upon death, so generally, the higher the density of infected insects in the environment, the higher the spore concentration. Since fungal infection in insects is achieved through spore transmission, spore concentration can be introduced into the model.

Summarize the reasons for the decrease in spore concentration in the environment as spore mortality and spore transfer caused by adhering to and infecting other insects. The decrease in spore concentration in the environment can be symbolically represented as the removal rate of spores in the environment, the reciprocal of which represents the incubation period of the fungal infectious disease.

Calculate the basic reproductive number of the model. The basic reproduction number can represent the average number of healthy insects infected per diseased insect after death during the infectious period. If the basic reproductive number is greater than 1, it indicates that on average. The spores produced by one infected insect during the incubation period can infect more than one healthy insect, and the disease caused by the initial small number of spores can spread within the insect population; otherwise, the disease will naturally die out. This usually requires the initial spore concentration in the environment to reach a threshold [99]. By altering the values of model parameters, simulating the epidemic trends under different conditions, and identifying the key factors influencing the spread of the disease, we made progress.

In the future, it is suggested to explore the relationship between different population densities and spore concentrations with the prevalence of fungal infection by controlling the model parameters. Research on the transmission laws of fungal diseases based on epidemiological models can provide scientific guidance and recommendations for the future use of fungi to control pests and manage diseases affecting beneficial insects.

## Figures and Tables

**Table 1:** The entomopathogenic fungi mentioned in this paper and their classification.

Species	Family	Order	Class	Phylum
*Ascosphaera apis*	*Ascosphaeraceae*	*Onygenales*	*Eurotiomycetes*	*Ascomycota*
*Ophiocordyceps unilateralis*	*Ophiocordycipitaceae*	*Hypocreales*	*Sordariomycetes*	*Ascomycota*
*Aschersonia aleyroids*	*Bionectriaceae*	*Hypocreales*	*Sordariomycetes*	*Ascomycota*
*Beauveria bassiana*	*Clavicipitaceae*	*Hypocreales*	*Sordariomycetes*	*Ascomycota*
*Metarhizium anisopliae*				
*Metarhizium rileyi*				
*Entomophthora muscae*	*Entomophthoraceae*	*Entomophthorales*	*Entomophthoromycetes*	*Entomophthoromycota*
*Massospora cicadina*				
*Eryniopsis lampyridarum*				
*Entomophaga grylli*				
*Pandora neoaphidis*				
*Batrachochytrium dendrobatidis*	*Batrachochytriaceae*	*Rhizophydiales*	*Chytridiomycota*	*Chytridiomycota*

The same background color indicates that these different species belong to the same Family, Order, Class, and Phylum.

## Data Availability

No new data were created or analyzed in this study.
